# 4-(5-Chloro­penta­namido)­benzene­sulfonamide

**DOI:** 10.1107/S1600536812048118

**Published:** 2012-11-28

**Authors:** Hasan Türkmen, Şerife Pınar Yalçın, Mehmet Akkurt, Mustafa Durgun

**Affiliations:** a Department of Chemistry, Faculty of Arts and Sciences, Harran University, 63300 Şanlıurfa, Turkey; b Department of Physics, Faculty of Arts and Sciences, Harran University, 63300 Şanlıurfa, Turkey; c Central Research Lab, Harran University, Osmanbey Campus, 63300 Şanlıurfa, Turkey; d Department of Physics, Faculty of Sciences, Erciyes University, 38039 Kayseri, Turkey

## Abstract

The mol­ecular conformation of the title compound, C_11_H_15_ClN_2_O_3_S, is stabilized by a C—H⋯O hydrogen bond, forming an *S*(6) ring motif. In the crystal, mol­ecules are linked by two pairs of inversion-related N—H⋯O hydrogen bonds, generating *R*_2_^2^(8) and *R*_2_^2^(20) ring motifs, resulting in chains running along [0-11]. These chains are connected by N—H⋯O hydrogen bonds along [100], forming layers parallel to (011). There are also C—H⋯π inter­actions between the layers, which consolidate the three-dimensional structure.

## Related literature

Sulfonamides represent an important class of biologically active compounds. For their action as inhibitors of carbonic anhydrase enzyme, their anti­bacterial properties in chemotherapy, as anti­thyroid drugs, and for their anti­microbial properties, see: Maren (1987 ▶); Supuran (2008 ▶); Turkmen *et al.* (2005 ▶, 2011 ▶); Rami *et al.* (2011 ▶). For their anti­viral properties, such as HIV protease inhibitors, see: De Clercq (2001 ▶) and as inhibitors of cysteine protease enzyme, see: Danial & Korsmeyer (2004 ▶). For related structures, see: Yalçın *et al.* (2012 ▶); Akkurt *et al.* (2010*a* ▶,*b* ▶). For hydrogen-bond motifs, see: Bernstein *et al.* (1995 ▶).
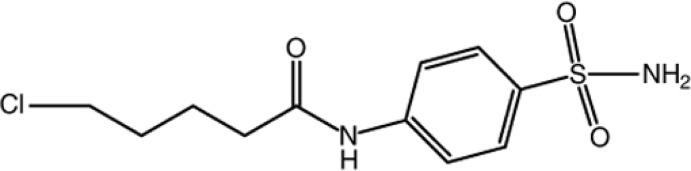


## Experimental

### 

#### Crystal data

C_11_H_15_ClN_2_O_3_S
*M* *_r_* = 290.77
Triclinic, 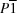

*a* = 8.4872 (1) Å
*b* = 8.7730 (2) Å
*c* = 10.4572 (3) Å
α = 73.711 (4)°
β = 85.281 (4)°
γ = 63.393 (3)°
*V* = 667.37 (3) Å^3^
*Z* = 2
Mo *K*α radiation
μ = 0.44 mm^−1^
*T* = 294 K
0.24 × 0.15 × 0.12 mm

#### Data collection

Rigaku R-AXIS RAPID-S diffractometer
Absorption correction: multi-scan (*SORTAV*; Blessing, 1995 ▶) *T*_min_ = 0.901, *T*_max_ = 0.949
20164 measured reflections
4036 independent reflections
2815 reflections with *I* > 2σ(*I*)
*R*_int_ = 0.068

#### Refinement

*R*[*F* ^2^ > 2σ(*F* ^2^)] = 0.065
*wR*(*F* ^2^) = 0.169
*S* = 1.05
4036 reflections
172 parameters
3 restraints
H atoms treated by a mixture of independent and constrained refinement
Δρ_max_ = 0.42 e Å^−3^
Δρ_min_ = −0.35 e Å^−3^

### 

Data collection: *CrystalClear* (Rigaku/MSC, 2005 ▶); cell refinement: *CrystalClear*; data reduction: *CrystalClear*; program(s) used to solve structure: *SIR97* (Altomare *et al.*, 1999 ▶); program(s) used to refine structure: *SHELXL97* (Sheldrick, 2008 ▶); molecular graphics: *ORTEP-3 for Windows* (Farrugia, 2012 ▶); software used to prepare material for publication: *WinGX* (Farrugia, 2012 ▶) and *PLATON* (Spek, 2009 ▶).

## Supplementary Material

Crystal structure: contains datablock(s) global, I. DOI: 10.1107/S1600536812048118/su2531sup1.cif

Structure factors: contains datablock(s) I. DOI: 10.1107/S1600536812048118/su2531Isup2.hkl

Supplementary material file. DOI: 10.1107/S1600536812048118/su2531Isup3.cml

Additional supplementary materials:  crystallographic information; 3D view; checkCIF report

## Figures and Tables

**Table 1:** Hydrogen-bond geometry (Å, °) *Cg*1 is the centroid of the C1–C6 ring.

*D*—H⋯*A*	*D*—H	H⋯*A*	*D*⋯*A*	*D*—H⋯*A*
C5—H5⋯O3	0.93	2.25	2.809 (4)	118
N1—H1*NA*⋯O3^i^	0.87 (2)	1.99 (3)	2.865 (3)	176 (3)
N1—H1*NB*⋯O1^ii^	0.87 (3)	2.11 (2)	2.963 (3)	166 (4)
N2—H2*N*⋯O2^iii^	0.88 (3)	2.17 (3)	3.021 (4)	166 (3)
C10—H10*A*⋯*Cg*1^iv^	0.97	2.96	3.771 (3)	142

Symmetry codes: (i) 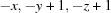; (ii) 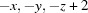; (iii) 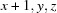; (iv) 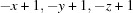.

**Fig. 1.**
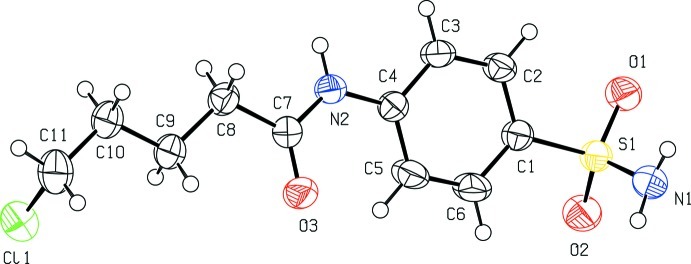
The view of the molecular structure of the title molecule, with the atom numbering. Displacement ellipsoids are drawn at the 50% probability level.

**Fig. 2.**
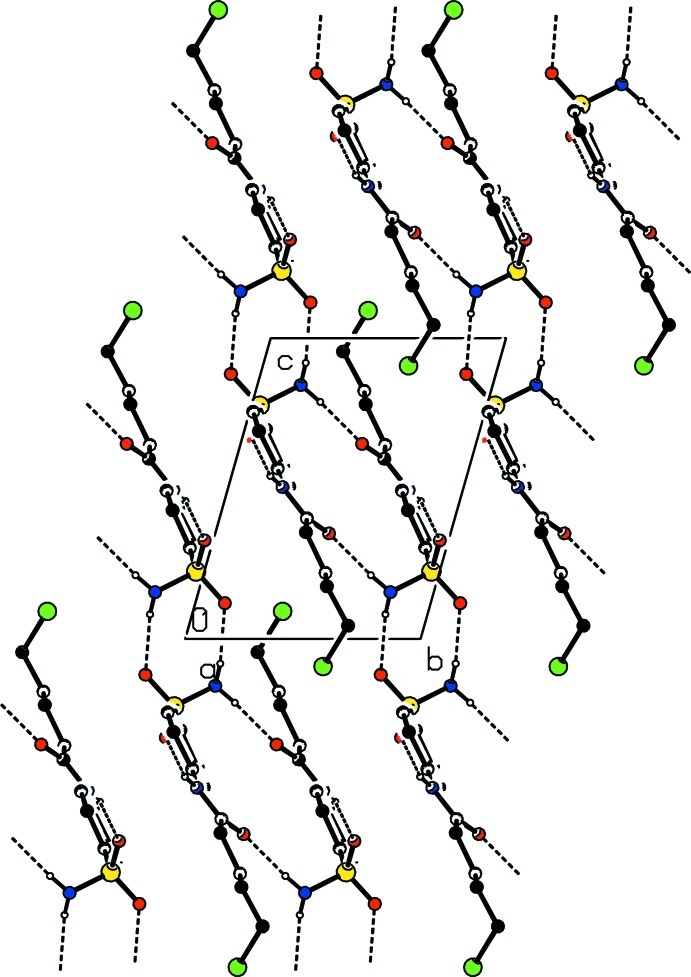
A partial view along the a axis of the crystal packing of the title compound, showing the inversion dimers formed by N—H···O hydrogen bonds. H atoms not involved in hydrogen bonding have been omitted for clarity.

## supplementary crystallographic information

### Comment

Sulfonamides represent an important class of biologically active compounds, with inhibitors of carbonic anhydrase enzyme, antibacterial properties in chemotherapy, antithyroid drugs, antimicrobial properties, (Maren, 1987; Rami *et al.*, 2011; Supuran, 2008; Turkmen *et al.*, 2005, 2011). Sulfonamides are also antiviral agents, such as as HIV protease inhibitors (De Clercq, 2001), and inhibitors of cysteine protease enzyme (Danial & Korsmeyer, 2004). The design and development of new sulfanilamide derivatives can help determine any structural requirements for improved biological activity. In this study, we have prepared and determined the crystal structure of the title compound.

In the 5-chloropentanamide moiety of the title compound, Fig. 1, the N2—C7—C8—C9, Cl1—C11—C10—C9 and O3—C7—C8—C9 torsion angles are 165.7 (2), 63.2 (3) and -16.5 (3) °, respectively. The bond lengths and bond angles are within the normal range and are comparable to those reported previously for the isomer 4-(3-chloro-2,2-dimethylpropanoylamino)-benzenesulfonamide (Yalçın *et al.*, 2012) and other related compounds (Akkurt *et al.*, 2010*a*,*b*).

A C—H···O hydrogen bond stabilizes the molecular conformation of the title molecule, forming a S(6) ring motif (Bernstein *et al.*, 1995; Table 1). In the crystal, neighbouring molecules are linked by two pairs of intermolecular N—H···O hydrogen bonds (Table 1 & Fig. 2), forming inversion dimers with *R*^2^_2_(8) and *R*^2^_2_(20) ring motifs, into chains running along [0 -1 1]. These chains are connected by N—H···O hydrogen bonds along the [100] direction, forming layers parallel to the (011) plane. C—H···π interactions between these layers further help in stabilizing the supramolecular structure (Table 1).

### Experimental

The title compound was prepared by a nucleophilic acyl substitution reaction of sulfanilamide with 5-chloropentanoylchloride. To a solution of 3.00 g (17.42 mmol) of sulfanilamide in 50.0 ml of THF, 4.41 ml (34.84 mmol) of NEM [N-ethylmaleimide] was added. A solution of 4.47 ml (34.84 mmol) of 5-chloropentanoylchloride in 20 ml of THF was added with stirring. A white precipitate of NEM.HCl salt was immediately observed. The reaction mixture was stirred at room temperature for 24 h, the progress of which was monitored by TLC (dichloromethane/methanol 6/1 *v*/*v*). The precipitate was filtered out and the filtrate collected was evaporated *in vacuo* to leave a residue. The residue was dissolved in ethyl acetate. The organic extract was washed with 3 *M* hydrochloric acid, then with saturated sodium bicarbonate solution and finally with brine. The extract was dried (MgSO_4_) and concentrated by evaporation *in vacuo* to give a residue. Recrystallization (ethanol) afforded 3.80 g (75%) the title compound as a white solid [*M*.p. 458–461 K]. Crystals suitable for X-ray diffraction were grown by slow evaporation of a solution in ethanol/chloroform/dichloromethane (4/3/3 *v*/*v*).

### Refinement

The H atoms on the NH and NH_2_ groups were located from a difference Fourier map and refined with distance restraints of N—H = 0.88 (1) Å, with *U*_iso_(H) = 1.2*U*_eq_(N). The C-bound H atoms were positioned geometrically, with C—H = 0.93 and 0.97 Å for CH and CH_2_ H atoms, respectively, and refined as riding with *U*_iso_(H) = 1.2*U*_eq_(C).

### Crystal data

**Table:**

C_11_H_15_ClN_2_O_3_S	*Z* = 2
*M**_r_* = 290.77	*F*(000) = 304
Triclinic, *P*1	*D*_x_ = 1.447 Mg m^−^^3^
Hall symbol: -P 1	Mo *K*α radiation, λ = 0.71073 Å
*a* = 8.4872 (1) Å	Cell parameters from 3036 reflections
*b* = 8.7730 (2) Å	θ = 2.7–30.5°
*c* = 10.4572 (3) Å	µ = 0.44 mm^−^^1^
α = 73.711 (4)°	*T* = 294 K
β = 85.281 (4)°	Needle, pale yellow
γ = 63.393 (3)°	0.24 × 0.15 × 0.12 mm
*V* = 667.37 (3) Å^3^	

### Data collection

**Table:**

Rigaku R-AXIS RAPID-S diffractometer	4036 independent reflections
Radiation source: Sealed Tube	2815 reflections with *I* > 2σ(*I*)
Graphite Monochromator monochromator	*R*_int_ = 0.068
Detector resolution: 10.0000 pixels mm^-1^	θ_max_ = 30.5°, θ_min_ = 2.7°
dtprofit.ref scans	*h* = −12→10
Absorption correction: multi-scan (*SORTAV*; Blessing, 1995)	*k* = −12→12
*T*_min_ = 0.901, *T*_max_ = 0.949	*l* = −14→14
20164 measured reflections	

### Refinement

**Table:**

Refinement on *F*^2^	Primary atom site location: structure-invariant direct methods
Least-squares matrix: full	Secondary atom site location: difference Fourier map
*R*[*F*^2^ > 2σ(*F*^2^)] = 0.065	Hydrogen site location: inferred from neighbouring sites
*wR*(*F*^2^) = 0.169	H atoms treated by a mixture of independent and constrained refinement
*S* = 1.05	*w* = 1/[σ^2^(*F*_o_^2^) + (0.0677*P*)^2^ + 0.2759*P*] where *P* = (*F*_o_^2^ + 2*F*_c_^2^)/3
4036 reflections	(Δ/σ)_max_ < 0.001
172 parameters	Δρ_max_ = 0.42 e Å^−^^3^
3 restraints	Δρ_min_ = −0.35 e Å^−^^3^

### Special details

**Table:**

Geometry. Bond distances, angles *etc*. have been calculated using the rounded fractional coordinates. All su's are estimated from the variances of the (full) variance-covariance matrix. The cell e.s.d.'s are taken into account in the estimation of distances, angles and torsion angles
Refinement. Refinement on *F*^2^ for ALL reflections except those flagged by the user for potential systematic errors. Weighted *R*-factors *wR* and all goodnesses of fit *S* are based on *F*^2^, conventional *R*-factors *R* are based on *F*, with *F* set to zero for negative *F*^2^. The observed criterion of *F*^2^ > σ(*F*^2^) is used only for calculating -*R*-factor-obs *etc*. and is not relevant to the choice of reflections for refinement. *R*-factors based on *F*^2^ are statistically about twice as large as those based on *F*, and *R*-factors based on ALL data will be even larger.

### Fractional atomic coordinates and isotropic or equivalent isotropic displacement parameters (Å^2^)

**Table:**

	*x*	*y*	*z*	*U*_iso_*/*U*_eq_	
Cl1	0.72069 (11)	0.61911 (12)	−0.09175 (8)	0.0692 (3)	
S1	−0.03920 (8)	0.03480 (8)	0.77585 (6)	0.0419 (2)	
O1	0.0449 (2)	−0.1257 (2)	0.88113 (18)	0.0500 (6)	
O2	−0.1417 (3)	0.0370 (3)	0.6723 (2)	0.0572 (7)	
O3	0.3519 (2)	0.4839 (2)	0.35111 (19)	0.0552 (6)	
N1	−0.1676 (3)	0.1842 (3)	0.8443 (2)	0.0535 (8)	
N2	0.5163 (3)	0.2301 (3)	0.5050 (2)	0.0416 (6)	
C1	0.1271 (3)	0.0902 (3)	0.6970 (2)	0.0398 (7)	
C2	0.2959 (3)	0.0145 (3)	0.7555 (2)	0.0426 (7)	
C3	0.4213 (3)	0.0629 (3)	0.6908 (2)	0.0434 (7)	
C4	0.3823 (3)	0.1871 (3)	0.5670 (2)	0.0385 (7)	
C5	0.2142 (4)	0.2581 (4)	0.5068 (3)	0.0586 (9)	
C6	0.0901 (4)	0.2088 (4)	0.5723 (3)	0.0611 (10)	
C7	0.4969 (3)	0.3716 (3)	0.4010 (2)	0.0389 (7)	
C8	0.6648 (3)	0.3843 (3)	0.3549 (3)	0.0424 (7)	
C9	0.6398 (3)	0.5164 (4)	0.2196 (3)	0.0479 (8)	
C10	0.8041 (4)	0.5387 (4)	0.1751 (3)	0.0481 (8)	
C11	0.7774 (5)	0.6762 (4)	0.0441 (3)	0.0614 (11)	
H1NA	−0.226 (4)	0.288 (2)	0.788 (3)	0.0830*	
H2	0.32410	−0.06880	0.83840	0.0510*	
H1NB	−0.123 (4)	0.179 (5)	0.918 (2)	0.0830*	
H2N	0.623 (2)	0.165 (4)	0.542 (3)	0.0830*	
H3	0.53400	0.01160	0.73060	0.0520*	
H5	0.18640	0.33860	0.42260	0.0700*	
H6	−0.02150	0.25670	0.53150	0.0730*	
H8A	0.75400	0.26840	0.35040	0.0510*	
H8B	0.70720	0.41920	0.41990	0.0510*	
H9A	0.60360	0.47760	0.15390	0.0570*	
H9B	0.54550	0.63050	0.22290	0.0570*	
H10A	0.84370	0.57130	0.24300	0.0580*	
H10B	0.89650	0.42580	0.16730	0.0580*	
H11A	0.88480	0.69000	0.02530	0.0740*	
H11B	0.68420	0.78900	0.05110	0.0740*	

### Atomic displacement parameters (Å^2^)

**Table:**

	*U*^11^	*U*^22^	*U*^33^	*U*^12^	*U*^13^	*U*^23^
Cl1	0.0689 (5)	0.0792 (6)	0.0525 (4)	−0.0331 (4)	0.0030 (4)	−0.0072 (4)
S1	0.0379 (3)	0.0438 (3)	0.0432 (3)	−0.0228 (3)	−0.0011 (2)	−0.0018 (2)
O1	0.0490 (10)	0.0413 (9)	0.0534 (10)	−0.0225 (8)	−0.0011 (8)	0.0015 (8)
O2	0.0533 (11)	0.0741 (13)	0.0549 (11)	−0.0404 (10)	−0.0057 (9)	−0.0101 (10)
O3	0.0387 (10)	0.0531 (11)	0.0557 (11)	−0.0175 (9)	−0.0005 (8)	0.0077 (9)
N1	0.0487 (14)	0.0476 (13)	0.0476 (13)	−0.0144 (11)	0.0006 (11)	−0.0003 (10)
N2	0.0343 (10)	0.0437 (11)	0.0411 (11)	−0.0180 (9)	−0.0022 (8)	−0.0009 (9)
C1	0.0373 (12)	0.0411 (12)	0.0416 (12)	−0.0213 (10)	−0.0008 (10)	−0.0047 (10)
C2	0.0409 (13)	0.0424 (12)	0.0399 (12)	−0.0211 (11)	−0.0056 (10)	0.0023 (10)
C3	0.0342 (12)	0.0422 (12)	0.0455 (13)	−0.0153 (10)	−0.0078 (10)	0.0000 (10)
C4	0.0357 (12)	0.0432 (12)	0.0378 (11)	−0.0203 (10)	0.0003 (9)	−0.0076 (10)
C5	0.0488 (15)	0.0720 (18)	0.0456 (14)	−0.0362 (14)	−0.0149 (12)	0.0185 (13)
C6	0.0435 (15)	0.077 (2)	0.0528 (15)	−0.0348 (15)	−0.0160 (12)	0.0159 (14)
C7	0.0376 (12)	0.0420 (12)	0.0365 (11)	−0.0177 (10)	0.0035 (10)	−0.0101 (10)
C8	0.0365 (12)	0.0432 (12)	0.0464 (13)	−0.0186 (10)	0.0051 (10)	−0.0100 (10)
C9	0.0426 (14)	0.0565 (15)	0.0474 (14)	−0.0287 (12)	0.0048 (11)	−0.0076 (12)
C10	0.0458 (14)	0.0527 (15)	0.0517 (14)	−0.0292 (12)	0.0095 (12)	−0.0126 (12)
C11	0.0676 (19)	0.0628 (18)	0.0634 (18)	−0.0410 (16)	0.0158 (15)	−0.0143 (15)

### Geometric parameters (Å, º)

**Table:**

Cl1—C11	1.797 (4)	C7—C8	1.508 (4)
S1—O1	1.4365 (18)	C8—C9	1.516 (4)
S1—O2	1.435 (2)	C9—C10	1.512 (5)
S1—N1	1.593 (2)	C10—C11	1.504 (4)
S1—C1	1.763 (3)	C2—H2	0.9300
O3—C7	1.220 (3)	C3—H3	0.9300
N2—C4	1.408 (4)	C5—H5	0.9300
N2—C7	1.356 (3)	C6—H6	0.9300
N1—H1NA	0.87 (2)	C8—H8A	0.9700
N1—H1NB	0.87 (3)	C8—H8B	0.9700
N2—H2N	0.88 (3)	C9—H9A	0.9700
C1—C2	1.390 (4)	C9—H9B	0.9700
C1—C6	1.377 (4)	C10—H10A	0.9700
C2—C3	1.377 (4)	C10—H10B	0.9700
C3—C4	1.389 (3)	C11—H11A	0.9700
C4—C5	1.397 (4)	C11—H11B	0.9700
C5—C6	1.374 (5)		
			
O1—S1—O2	118.85 (13)	C1—C2—H2	120.00
O1—S1—N1	106.87 (11)	C3—C2—H2	120.00
O1—S1—C1	107.57 (11)	C2—C3—H3	120.00
O2—S1—N1	107.06 (14)	C4—C3—H3	119.00
O2—S1—C1	106.58 (13)	C4—C5—H5	120.00
N1—S1—C1	109.75 (13)	C6—C5—H5	120.00
C4—N2—C7	127.3 (2)	C1—C6—H6	119.00
S1—N1—H1NB	114 (3)	C5—C6—H6	119.00
H1NA—N1—H1NB	119 (3)	C7—C8—H8A	109.00
S1—N1—H1NA	114.0 (18)	C7—C8—H8B	109.00
C7—N2—H2N	115.5 (19)	C9—C8—H8A	109.00
C4—N2—H2N	116.9 (18)	C9—C8—H8B	109.00
S1—C1—C6	119.1 (2)	H8A—C8—H8B	108.00
S1—C1—C2	121.99 (17)	C8—C9—H9A	109.00
C2—C1—C6	118.9 (3)	C8—C9—H9B	109.00
C1—C2—C3	120.0 (2)	C10—C9—H9A	109.00
C2—C3—C4	121.1 (2)	C10—C9—H9B	109.00
C3—C4—C5	118.6 (3)	H9A—C9—H9B	108.00
N2—C4—C5	122.8 (2)	C9—C10—H10A	109.00
N2—C4—C3	118.6 (2)	C9—C10—H10B	109.00
C4—C5—C6	119.8 (3)	C11—C10—H10A	109.00
C1—C6—C5	121.5 (3)	C11—C10—H10B	109.00
O3—C7—C8	122.4 (2)	H10A—C10—H10B	108.00
N2—C7—C8	115.8 (2)	Cl1—C11—H11A	109.00
O3—C7—N2	121.8 (3)	Cl1—C11—H11B	109.00
C7—C8—C9	112.6 (2)	C10—C11—H11A	109.00
C8—C9—C10	113.4 (2)	C10—C11—H11B	109.00
C9—C10—C11	113.6 (3)	H11A—C11—H11B	108.00
Cl1—C11—C10	112.5 (3)		
			
O1—S1—C1—C2	−15.8 (2)	C6—C1—C2—C3	2.2 (4)
O2—S1—C1—C2	−144.3 (2)	C1—C2—C3—C4	0.0 (4)
N1—S1—C1—C2	100.2 (2)	C2—C3—C4—N2	−179.4 (2)
O1—S1—C1—C6	162.6 (2)	C2—C3—C4—C5	−2.2 (4)
O2—S1—C1—C6	34.2 (3)	N2—C4—C5—C6	179.2 (3)
N1—S1—C1—C6	−81.5 (2)	C3—C4—C5—C6	2.1 (4)
C7—N2—C4—C3	−164.6 (2)	C4—C5—C6—C1	0.1 (5)
C7—N2—C4—C5	18.3 (4)	O3—C7—C8—C9	−16.5 (3)
C4—N2—C7—O3	2.2 (4)	N2—C7—C8—C9	165.7 (2)
C4—N2—C7—C8	180.0 (2)	C7—C8—C9—C10	176.7 (2)
S1—C1—C2—C3	−179.42 (18)	C8—C9—C10—C11	−177.1 (3)
S1—C1—C6—C5	179.3 (2)	C9—C10—C11—Cl1	−63.2 (3)
C2—C1—C6—C5	−2.2 (4)		

### Hydrogen-bond geometry (Å, º)

Cg1 is the centroid of the C1–C6 ring.

**Table:**

*D*—H···*A*	*D*—H	H···*A*	*D*···*A*	*D*—H···*A*
C5—H5···O3	0.93	2.25	2.809 (4)	118
N1—H1*NA*···O3^i^	0.87 (2)	1.99 (3)	2.865 (3)	176 (3)
N1—H1*NB*···O1^ii^	0.87 (3)	2.11 (2)	2.963 (3)	166 (4)
N2—H2*N*···O2^iii^	0.88 (3)	2.17 (3)	3.021 (4)	166 (3)
C10—H10*A*···*Cg*1^iv^	0.97	2.96	3.771 (3)	142

Symmetry codes: (i) −*x*, −*y*+1, −*z*+1; (ii) −*x*, −*y*, −*z*+2; (iii) *x*+1, *y*, *z*; (iv) −*x*+1, −*y*+1, −*z*+1.
